# Seizures in Primary Antiphospholipid Syndrome: The Relevance of Smoking to Stroke

**DOI:** 10.1155/2012/981519

**Published:** 2012-03-25

**Authors:** Jozélio Freire de Carvalho, Sandra Gofinet Pasoto, Simone Appenzeller

**Affiliations:** ^1^Rheumatology Division, Hospital das Clínicas da Faculdade de Medicina da Universidade de São Paulo, 01246-903 São Paulo, SP, Brazil; ^2^Rheumatology Division, Hospital das Clínicas da Faculdade de Medicina da Universidade de Campinas, Campinas, SP, Brazil

## Abstract

*Objectives*. To evaluate the frequency of seizures in primary antiphospholipid syndrome (PAPS) and their possible clinical and laboratory associations. *Methods*. Eighty-eight PAPS patients (Sydney's criteria) were analyzed by a standard interview, physical examination and review of medical charts. Risk factors for seizures, clinical manifestations, associated comorbidities, and antiphospholipid antibodies were evaluated. *Results*. Nine (10.2%) patients with seizures were identified, 77.8% had convulsions onset after PAPS diagnosis. Mean age, gender, and race were comparable in groups with or without seizures. Interestingly, a higher frequency of current smoking (44.4 versus 10.1%, *P* = 0.019) was observed in the first group. Stroke, Sneddon's syndrome, and livedo reticularis were more frequent in PAPS patients with seizures than those without seizures, although not statistically significant (*P* > 0.05). Comparison between patients with seizures onset after PAPS diagnosis (*n* = 7) and those without convulsions (*n* = 79) demonstrated a higher frequency of current smoking (42.9 versus 10%, *P* = 0.042) and stroke in the first group (71.4 versus 30.4%, *P* = 0.041). Regression analysis confirmed that smoking (*P* = 0.030) and stroke (*P* = 0.042) were independently associated to seizures. *Conclusion*. About 10.2% of PAPS patients had convulsions, predominantly after PAPS diagnosis, and seizures were associated to current smoking and stroke.

## 1. Introduction

Antiphospholipid syndrome (APS) is a systemic autoimmune disorder characterized by the presence of arterial or venous thrombosis and/or recurrent fetal loss, in addition to serum antiphospholipid (aPL) antibodies, mainly lupus anticoagulant (LA) and anticardiolipin antibody (aCL) [1]. 

APS can occur in persons without an underlying systemic autoimmune disease (primary APS or PAPS) or in the presence of other systemic autoimmune diseases, particularly systemic lupus erythematosus (SLE) [1–3].

The frequency of aPL ranges from 1 to 10% in the general population; however, the exact prevalence of PAPS is not known. Based on a limited number of uncontrolled and non-risk-stratified studies, asymptomatic aPL-positive patients have 0 to 4% risk of annual thrombosis [4].

 In the arterial territory, the central nervous system (CNS) is the most affected organ, up to 22% of PAPS patients, usually in the form of stroke or transient ischemic attacks (TIA). Additional CNS manifestations described in these patients are, chorea, migraine, transverse myelitis, cognitive impairment and epileptic seizures [5–7].

The association between the presence of aPL and epileptic seizures has been studied in both animal models [8] and in large cohort studies, especially in association with SLE patients [9]. Previous studies suggested that aPL may have a direct effect on seizure genesis by increasing neuronal excitability through inhibition of *γ*-aminobutyric acid receptor-ion channel complex [10] or as consequence of antibody binding to neurons [8]. Other studies concluded that epileptic seizures in patients with aPL may be the expression of ischemic events secondary to hypercoagulability [11, 12].

However, there are no studies evaluating seizure frequency specifically in PAPS patients. Therefore we sought to determine the frequency and risk factors for epileptic seizures in a cohort of PAPS. We also analyzed clinical and laboratory features associated with the occurrence of single episodes and recurrence of epileptic seizures.

## 2. Methods

### 2.1. Patients

The records of 320 APS patients followed regularly at the Antiphospholipid Syndrome Outpatient Clinic of the Rheumatology Division, Hospital das Clínicas da Faculdade de Medicina da Universidade de São Paulo, were reviewed. Patients who had secondary causes of APS, such as SLE, rheumatoid arthritis, or vasculitis were excluded from this study. Furthermore, no selected patient had specific antibodies for SLE, anti-double-stranded DNA, or anti-Sm antibodies. Patients with seizures secondary to acute metabolic causes such as uremia, hypertension, diabetes mellitus, and electrolytic abnormalities were excluded. Therefore 88 patients (mean age 40.6 ± 11.1 years; 91% women) with PAPS, according to Sydney's criteria [13] were included. All subjects provided informed consent, and the study was approved by the local Ethical Committee.

### 2.2. Demographic and Clinical Data, Comorbidities, and Medications

Demographic, clinical, and therapeutic data were obtained through a standard interview, physical examination, and exhaustive review of medical charts. The date of PAPS diagnosis, as well as the onset of epileptic seizures, was registered. Variables collected included age, gender, race, weight, height, and body mass index (BMI) (weight/height^2^). Clinical and therapeutic data, including disease duration, thrombotic and obstetric manifestations, stroke, acute myocardial infarction, chronic comorbidities such as hypertension, diabetes, dyslipidemia, metabolic syndrome, current or previous history of smoking, alcoholism, and medication history of warfarin, heparin, aspirin, anticonvulsants, and antimalarial drugs were obtained from the medical records and interviews. Diabetes mellitus was defined as fasting blood glucose >126 mg/dL or current use of insulin and/or oral hypoglycemic drugs. Hypertension was defined as systolic blood pressure >140 mmHg or diastolic blood pressure >90 mmHg or current use of antihypertensive drugs [14]. Metabolic syndrome and dyslipidemia were defined according to the International Diabetes Federation (IDF) consensus [15].

### 2.3. Neurologic Evaluation

Seizures were classified according to the International League Against Epilepsy criteria [16, 17]. Electroencephalograms (EEGs) were recorded in the interictal period in a 16-channel analog or 32-channel digital EEG recorder with the International 10–20 System of electrode placement for 20 to 30 minutes. We tabulated the presence and localization of interictal epileptiform abnormality and slow wave abnormality. Magnetic resonance imaging (MRI) was performed in all patients who had seizures or other CNS manifestations [18].

### 2.4. Autoantibodies

Serum IgG and IgM anticardiolipin (aCL) antibodies were measured at least twice with an interval of 6 weeks by ELISA as previously described [19], using a commercially available kit (Quanta Lite ACA, INOVA Diagnostics, USA). According to the manufacturer's recommendations and Sydney criteria [13], only titers of aCL ≥40 GPL or MPL were considered positive. Lupus anticoagulant was measured according to international guidelines using activated partial thromboplastin time (APTT, Diagnostica Stago, France) and diluted Russell's viper venom time (dRVVT, Trinity Biotech, Wicklow, Ireland) [20]. IgG and IgM anti-beta2-glycoprotein were measured once using a commercial ELISA kit according to the manufacturer's instructions (Quanta Lite, INOVA Diagnostics Inc, CA, USA). As described in the kit, the positive standard used to construct the calibration curve came from the Rheumatology Lab, Seton Hall University, St. Joseph's Hospital and Medical Center. Only titers of ACL and anti-beta2-glycoprotein >40 U/mL were considered positive.

In order to exclude systemic lupus erythematosus and other connective diseases such as Sjogren's syndrome, we assessed the following antibodies: anti-double-stranded DNA (anti-dsDNA) antibodies were detected by indirect immunofluorescence using as substrates *Crithidia luciliae *[21]. Hemagglutination assay with rabbit thymus extract was used to determine the presence of antibodies to RNP and Sm proteins [22].

### 2.5. Statistical Analysis

Evaluation was performed using the computer program GraphPad. Results are shown as mean ± standard deviation or percentage. Chi-square, Fisher's exact, Student's *t*- and Mann-Whitney tests were used for comparisons between both groups of patients, with or without seizures, when applicable. Logistic regression was used to analyze associations between seizures and the several evaluated features (dependent variable). *P* values < 0.05 were considered to be statistically significant. 

## 3. Results

Seizures were identified in 9 (10.2%) of 88 PAPS patients. Two of them (22.2%) had seizures onset before and 7 (77.8%) patients after the diagnosis of PAPS. Five of nine (55.6%) patients had partial complex seizures, 3 (33.3%) had generalized tonic-clonic seizures, and 1 (11.1%) had focal seizure. Interictal EEG was done in 7/9 patients and showed epileptiform activity in 6 patients. MRI was done in all patients, and the majority (6/9) of the patients had evidence of ischemic lesions (Table 1). 

### 3.1. Comparative Analysis between PAPS Patients with or without Seizures

Current smoking was more observed in PAPS patients with seizures than those without this neurological abnormality (44.4 versus 10.1%; *P* = 0.019). In addition, previous history of smoking was also more frequently observed in PAPS with seizures when compared to the other patients (66.7 versus 35.4%, *P* = 0.083), although it did not reach statistical significance. Similarly, stroke (66.7 versus 30.4%, *P* = 0.057), Sneddon's syndrome (44.4 versus 15.2%, *P* = 0.053), and livedo reticularis (66.7 versus 30.4%, *P* = 0.057) had a trend to be more frequently observed in PAPS patients with epileptic seizures. No difference regarding other clinical APS manifestations, disease duration, risk factors for cerebrovascular diseases, medications, and antiphospholipid antibodies was observed between the groups (*P* > 0.05) (Table 2). 

### 3.2. Comparative Analysis between Patients with Onset of Seizures after PAPS Diagnosis and Those without Seizures

The comparison of the 7 patients with onset of seizures after PAPS diagnosis and those without seizures (*n* = 79) demonstrated a higher frequency of current smoking (42.9 versus 10.1%, *P* = 0.042) and stroke (71.4 versus 30.4%, *P* = 0.041) in the first group (Table 3). Regression analysis revealed that smoking (OR: 7.37, 95% CI: 1.21–44.83, *P* = 0.030) and stroke (OR: 6.5, 95% CI: 1.07–39.44, *P* = 0.042) were independent variables associated to seizures. 

### 3.3. Comparative Analysis between Patients with Single and Recurrent Epileptic Seizures

Four of 9 (44.4%) patients had recurrent seizures and 5 (55.6%) had a single seizure. Patients with recurrent seizures had higher levels of IgG anticardiolipin (95 (32–120) versus 20 (0–74) GPL, *P* = 0.035) and less frequently livedo reticularis (25 versus 100%, *P* = 0.048) than patients with a single seizure. The other parameters were alike between the groups (*P* > 0.05). 

## 4. Discussion

In this group of 88 PAPS patients, 9 (10.2%) had seizures. The frequency of seizures in previous studies was about 7% [5–7]. Generalized tonic-clonic and complex focal seizures were the most common seizures observed in this study. 

Current smoking was the only feature associated with seizures in our PAPS patients with this neurological manifestation. Similar results were observed in a large cohort of healthy women studied prospectively over 15 years after adjustment for stroke and other potential confounding factors [23]. One possible biologic explanation is a direct effect of nicotine, which is an excitatory neurotransmitter that enhances glutamate release. In predisposed mice, nicotine has dose-dependent convulsive effects: at low doses, the onset of seizures was delayed [24], whereas at high doses, nicotine caused convulsions [25]. Nicotine is also a stimulant and increases blood pressure and impairs sleep. In addition, smoking causes tissue hypoxia due to chronic carbon monoxide exposure [26]. All these factors may indirectly lead to seizures. Unfortunately, we did not have data regarding nicotine dosage in our patients. However, since the presence of aPL increases neuronal excitability, the association with nicotine may represent a risk factor for seizure genesis in these patients [25]. PAPS patients should therefore strongly be discouraged from nicotine abuse. 

 Seizures before PAPS diagnosis were identified in 2 out of 9 patients. Although we do not know if these patients had positive aPL before the thrombotic event, we did not exclude these patients because autoantibodies have been shown to appear in the systemic circulation years before the onset of autoimmune diseases [27]. 

 Seizures occurred after PAPS diagnosis in 7/9 (77.8%) patients. Both current smoking and stroke were independently associated with seizures in these patients. Previous studies have identified stroke as a risk factor for seizures in APS and SLE [9, 18]. Furthermore, most patients had evidence of ischemic lesions on MRI. These findings suggest that PAPS patients with new onset of seizures should be screened for ischemic cerebral lesions, independently of their previous treatment for APS. In addition, it is well known that APS patients are prone to atherosclerosis due to several cardiovascular factors [28, 29]. 

Single seizures were observed in 5 of 9 patients. Interestingly, livedo reticularis was more frequently observed in these patients when compared to patients with recurrent epileptic seizures. This finding is difficult to explain, since livedo reticularis is commonly linked to stroke, and this neurological event was positively associated with seizures after APS onset in our patients. 

In PAPS patients with recurrent epileptic seizures, we observed significant higher IgG anticardiolipin levels when compared to patients with single seizures episodes, reinforcing the role of aPL in seizure genesis. The association between higher titers of antiphospholipid antibodies and seizures has been previously demonstrated [8, 10].

Different from previous studies, this paper evaluated the frequency of seizures and their risk factors specifically in primary APS according to the currently accepted criteria [13]. Indeed, it is known that in SLE, the main disease associated to secondary APS, there are multiple causes for seizures, such as nervous central system activity, vasculitis, stroke, and meningoencephalitis [9, 30]. Moreover, all selected patients were negative for lupus-specific autoantibodies. Other additional benefit is the overview analysis of several risk factors for convulsions, allowing a more accurate evaluation of the relevance of each risk factor for convulsions. 

In conclusion, we observed the occurrence of seizures in 10.2% of patients with PAPS, which occurred predominantly after the diagnosis of this disorder, and were independent factor associated with current smoking and stroke. These findings suggest that the pathogenic mechanism of seizures in PAPS is consequent to ischemic cerebral events in addition to excitatory mechanism of both nicotine and aPL. All PAPS patients with new onset of seizures should be screened for ischemic cerebral events. Furthermore, our data reinforce the need for smoking cessation, as well as rigorous control of cardiovascular risk factors in these patients. 

## 5. Key Messages

1. About 10.2% of patients with primary antiphospholipid syndrome have seizures.
2. Seizures in primary antiphospholipid syndrome occur mainly after the APS diagnosis and are linked to current smoking and stroke.
3. Treatment of seizures in APS includes the need for smoking cessation, as well as rigorous control of cardiovascular risk factors in these patients.

## Figures and Tables

**Table 1:** Characteristics of the 9 PAPS patients with epileptic seizures.

	Thrombotic event	Comorbidity	Age (years) sex, race	Seizure onset after PAPS	LR	Seizure type	>1 seizure	MRI	EEG	Drugs
1	DVT	Hypothyroidism	49, F, C	−	+	Generalized tonic-clonic	+	NL	EA in frontal region	CBZ, PHT, PB, WAR
2	Stroke	Hypertension	52, F, C	+	+	Partial complex	−	Right fronto-parietooccipital stroke	NL	PB, ASA
3	Stroke	Chorea gravidarum, preeclampsia	29, F, C	+	−	Partial complex with secondary generalization	+	Left frontal and basal ganglia hyperintense lesions	EA in left parietooccipital region	PB, PHT, CLB, GBP, ASA
4	DVT, PTE, stroke, miscarriages	Rheumatic fever	32, F, C	+	+	Partial	+	Right temporo-parietooccipital stroke	EA in right parietal region	CLB, PHT, TPM, WAR
5	DVT, miscarriages	Depression	51, F, Mul	−	−	Partial complex	+	Left periventricular and parietal stroke	EA in left parietal region	CBZ, OXC, WAR
6	Renal TMA, stroke, TIA, miscarriages	Renal transplantation depression	53, F, C	+	−	Partial complex with secondary generalization	−	Left frontotemporal srtroke	EA in left temporal region	PHT, WAR, ASA
7	Thrombocytopenia, stroke	Hypothyroidism splenectomy	45, M, C	+	−	Partial complex with secondary generalization	−	Stroke of territory of the middle cerebral artery	EA in left temporal region	CBZ, ASA
8	DVT, thrombocytopenia	—	35, M, C	+	−	Generalized tonic-clonic	−	NL	ND	PB, PHT, WAR
9	DVT, thrombocytopenia	Compulsive gambler	53, M, C	+	+	Generalized tonic-clonic	−	NL	ND	PHT, WAR

ASA: aspirin; CBZ: carbamazepine; C: Caucasian; CLB: clobazam; DVT: deep venous thrombosis; EA: epileptiform activity; EEG: electroencephalogram; F: female; GBP: gabapentin; M: male; Mul: mulatto; NL: normal; ND: not done; PAPS: primary antiphospholipid syndrome; PTE: pulmonary thromboembolism; TMA: microangiopathic; OXC: oxcarbazepine; PB: phenobarbital; PHT: phenytoin; TPM: topiramate; WAR: warfarin.

**Table 2:** Comparisons of demographic, clinical, and laboratory features, vascular risk factors, and medications between the 9 PAPS patients with seizures and PAPS patients without seizures (*n* = 79).

	PAPS with seizures	PAPS without seizures	*P* values
	*n* = 9	*n* = 79	
Age, years	44.6 ± 10.7	40.1 ± 11.2	0.261
White race, *n* (%)	7 (77.8)	65 (82.3)	0.665
Female gender, *n* (%)	7 (77.8)	65 (82.3)	0.665
Body mass index, kg/m^2^	27.8 ± 3.1	28.0 ± 7.5	0.933
Arterial events, *n* (%)	6 (66.7)	38 (48.1)	0.484
Venous events, *n* (%)	4 (44.4)	49 (62.0)	0.474
Obstetric events, *n* (%)	3 (33.3)	31 (39.2)	1.000
Thrombocytopenia, *n* (%)	2 (22.2)	19 (24.1)	1.000
Livedo reticularis, *n* (%)	6 (66.7)	24 (30.4)	0.057
Stroke, *n* (%)	6 (66.7)	24 (30.4)	0.057
Sneddon's syndrome, *n* (%)	4 (44.4)	12 (15.2)	0.053
Pulmonary thromboembolism, *n* (%)	1 (11.1)	19 (24.1)	0.678
Deep venous thrombosis, *n* (%)	4 (44.4)	40 (50.6)	0.680
Angina, *n* (%)	1 (11.1)	7 (8.9)	1.000
Acute myocardial infarction, *n* (%)	0	1 (1.2)	1.000
Sedentarism, *n* (%)	6 (66.7)	50 (63.3)	1.000
Disease duration, months	88.4 ± 94	94.6 ± 64.3	0.398
Metabolic syndrome, *n* (%)	0	17 (21.5)	0.270
Diabetes, *n* (%)	0	6 (7.6)	0.874
Systemic hypertension, *n* (%)	4 (44.4)	37 (46.8)	0.892
Previous history of smoking, *n* (%)	5 (55.5)	28 (35.4)	<0.001
Current smoking, *n* (%)	4 (44.4)	8 (10.1)	0.019
Alcoholism, *n* (%)	1 (11.1)	0	0.102
Heparin use, *n* (%)	6 (66.7)	40 (50.6)	0.575
Current chloroquine use, *n* (%)	2 (22.2)	37 (46.8)	0.153
Statin use, *n* (%)	4 (44.4)	20 (25.3)	0.680
IgG anticardiolipin levels, GPL	55.1 ± 45.9	40.0 ± 46.2	0.357
IgG anticardiolipin, *n* (%)	7 (77.8)	44 (55.7)	0.293
IgM anticardiolipin levels, MPL	17.7 ± 19.9	29.9 ± 40.2	0.373
IgM anticardiolipin, *n* (%)	5 (55.6)	40 (50.6)	1.000
Lupus anticoagulant, *n* (%)	6 (66.7)	60 (75.9)	0.839
IgG anti-beta2-glycoprotein I, U/mL	26.6 ± 31.32	22.6 ± 35.02	0.809
IgG anti-beta2-glycoprotein I, *n* (%)	2 (22.2)	13 (20.9)	0.669
IgM anti-beta2-glycoprotein I, U/mL	12.0 ± 12.4	14.5 ± 32.4	0.862
IgM anti-beta2-glycoprotein I, *n* (%)	1 (11.1)	7 (8.9)	1.000

Data expressed as mean ± standard deviation or percentage; PAPS: primary antiphospholipid syndrome; *n* = number of patients; SD = standard deviation.

**Table 3:** Comparisons of demographic, clinical, and laboratory features, vascular risk factors, and medications between the 7 PAPS patients with seizures after APS diagnosis and those without seizures (*n* = 79).

	PAPS with epileptic seizures after APS onset *n* = 7	PAPS without epileptic seizures *n* = 79	*P* value
Age, years	42 ± 10.3	40.1 ± 11.2	0.670
White race, *n* (%)	5 (71.4)	65 (82.3)	0.610
Female gender, *n* (%)	5 (71.4)	65	0.610
Body mass index, kg/m^2^	27.5 ± 2.9	28.0 ± 7.5	0.866
Arterial events, *n* (%)	5 (71.4)	38 (48.1)	0.433
Venous events, *n* (%)	2 (28.6)	49 (62.0)	0.115
Obstetric events, *n* (%)	2 (28.6)	31 (39.2)	0.703
Thrombocytopenia, *n* (%)	2 (28.6)	19 (24.1)	1.000
Livedo reticularis, *n* (%)	4 (57.1)	24 (30.4)	0.208
Stroke, *n* (%)	5 (71.4)	24 (30.4)	0.041
Sneddon's syndrome, *n* (%)	3 (42.9)	12 (15.2)	0.098
Pulmonary thromboembolism, *n* (%)	1 (14.3)	19 (24.1)	0.678
Deep venous thrombosis, *n* (%)	2 (28.6)	40 (50.6)	0.434
Angina, *n* (%)	0	7 (8.9)	1.000
Acute myocardial infarction, *n* (%)	0	1 (1.3)	1.000
Sedentarism, *n* (%)	5 (71.4)	50 (63.3)	1.000
Disease duration, months	92.6 ± 101.9	94.6 ± 64.3	0.470
Metabolic syndrome, *n* (%)	0	17 (21.5)	0.336
Diabetes, *n* (%)	0	6 (7.6)	1.000
Systemic hypertension, *n* (%)	3 (42.9)	37 (46.8)	1.000
Previous history of smoking, *n* (%)	5 (71.4)	28 (35.4)	0.101
Current smoking, *n* (%)	3 (42.9)	8 (10.1)	0.042
Alcoholism, *n* (%)	1 (14.3)	0	1.000
Heparin use, *n* (%)	4 (57.1)	37 (46.8)	1.000
Current chloroquine use, *n* (%)	2 (28.6)	37 (46.8)	0.448
Statin use, *n* (%)	0	20 (25.3)	0.193
IgG anticardiolipin levels, GPL	57.4 ± 50.4	40.0 ± 46.3	0.347
IgG anticardiolipin, *n* (%)	5 (71.4)	44 (55.7)	1.000
IgM anticardiolipin levels, MPL	19.9 ± 21.6	29.9 ± 40.2	0.518
IgM anticardiolipin, *n* (%)	4 (57.1)	40 (50.6)	1.000
Lupus anticoagulant, *n* (%)	5 (71.4)	60 (75.9)	1.000

Data expressed as mean ± standard deviation or percentage; PAPS: primary antiphospholipid syndrome; *n* = number of patients; SD = standard deviation.
